# Large nuclear cardiology laboratory slashes radiation dose by 60% in eight years

**Published:** 2018

*A large nuclear cardiology laboratory has slashed its average radiation dose by 60% in eight years, according to new research* presented recentlyat the International Conference on Nuclear Cardiology (ICNC) 2017 and published in J Am Coll Cardiol: Cardiovasc Imaging. The study in over 18 000 patients shows dose reductions were achieved despite a large number of obese patients.

‘There has been concern among the medical community and the public that the radiation from medical diagnostic tests could increase the risk of cancer, said Professor Randall Thompson, a cardiologist at the Mid-America Heart Institute, Kansas City, Missouri, US.

He continued: ‘Although the risk of harm from an individual nuclear cardiology test is very low – even very conservative estimates suggest only one in 1 000 extra patients would develop cancer 20 years later – the cumulative dose from multiple medical diagnostic tests may be a concern.’

Medical societies advocate getting radiation doses as low as is reasonably achievable. There are ways to do this but surveys show that adoption of new technologies, which cost money, and new testing algorithms, which take more physician time, has been slow.

This study assessed the impact on radiation dose of modifying protocols and introducing new hardware (cameras) and post-processing software in a large nuclear cardiology laboratory network in Kansas City.

The study included the 18 162 single-photon emission computed tomography (SPECT) myocardial perfusion imaging (MPI) studies performed at all four of the Saint Luke’s Mid-America Heart Institute nuclear cardiology laboratories from 1 January 2009 to 30 September 2016. SPECT MPI shows how well blood flows through the muscle of the heart and is primarily performed to diagnose the cause of chest pain or to help manage patients with known coronary artery disease.

Protocols were modified by performing stress-only tests where possible, which saves the radiotracer dose from the rest scan. Stress and rest scans are still required in some patients since shadowing from body parts can look like a lack of blood flow and two scans can clarify the findings. Technetium tracers are now used instead of thallium 100% of the time at one-third of the radiation dose.

Small field-of-view cameras, which have advanced post processing, and a new generation of camera systems, which are more sensitive and need less radiotracer injected into the body, have both been introduced. These camera systems are equipped with advanced processing which enhances the nuclear pictures and need less radiation or shorter image acquisition times. Professor Thompson’s laboratory focused primarily on reducing the radiation dose.

The average radiation dose fell from 17.9 mSv in 2009 to 7.2 mSv in 2016 and the median dose (the 50th percentile) dropped from 10.2 to 2.5 mSv. Professor Thompson said: ‘There was a dramatic lowering of the radiation dose with all of these concerted efforts. The average dose fell by 60% and the median dropped by 75%.’

‘The average dose had fallen to 5.4 mSv in 2012 but crept up as we’ve had more obese patients referred in whom we have to use the higher dose protocols,’ he added. ‘But more than half of patients now are tested with a low-dose, stressonly test using the new technology, which is why the median dose of radiation has fallen so dramatically.’

The average background dose for people living in Europe and North America from radon underground and cosmic background sources is about 3 mSv a year. Medical societies consider higher- and lower-dose tests to be above 10 mSv and below 3 mSv, respectively. In 2010 the American Society of Nuclear Cardiology set a target of 9 mSv or less for the majority of tests.

Professor Thompson said: ‘The majority of studies were in the high-dose range back in 2009 and now most tests have a radiation dose that is about a third of the target. This is despite being referred a larger number of obese patients. In the last 2.5 years, 17% of patients have needed the large fieldof- view camera as their average body mass index was 46 kg/ m2 and they were simply too big for the small cameras.’

He concluded: ‘By adopting contemporary protocols and technologies it is feasible to substantially lower radiation doses in nuclear cardiology in very large numbers of patients in real world clinical practice.’
